# Supplementary material for: Thermodynamic dissipation constrains metabolic versatility of unicellular growth
Source: Nat Commun. 2025 Sep 29;16:8543. doi: 10.1038/s41467-025-62975-5 (PMC12480764; doi:10.1038/s41467-025-62975-5)
Supplement: Supplementary file 2 — Description of Additional Supplementary Information [file 41467_2025_62975_MOESM2_ESM.pdf]

Description of Additional Supplementary Files

File Name: Supplementary Data 1
Description: experimentally measured yields parsed from primary sources.

File Name: Supplementary Data 2
Description: yields of biomass and of metabolic substrates and products.

File Name: Supplementary Data 3
Description: auxiliary information on the experiments parsed from primary sources.

File Name: Supplementary Data 4
Description: stoichiometric composition of biomass.

File Name: Supplementary Data 5
Description: thermodynamic properties of chemicals in the dataset.

File Name: Supplementary Data 6
Description: main thermodynamic quantities computed.

File Name: Supplementary Data 7
Description: bibliography for the Supplementary Data files.
