# Supplementary material for: Thermodynamic dissipation constrains metabolic versatility of unicellular growth
Source: Nat Commun. 2025 Sep 29;16:8543. doi: 10.1038/s41467-025-62975-5 (PMC12480764; doi:10.1038/s41467-025-62975-5)
Supplement: Supplementary file 9 — Supplementary Data 7 [file 41467_2025_62975_MOESM9_ESM.pdf]

- Heijnen and Kleerebezem (2010): Joseph J Heijnen and Robbert Kleerebezem. Bioenergetics of microbial growth. *Encyclopedia of Industrial Biotechnology: Bioprocess, Bioseparation, and Cell Technology*, pages 1–66, 2009;

  Robbert Kleerebezem and Mark CM Van Loosdrecht. A generalized method for thermodynamic state analysis of environmental systems. *Critical Reviews in Environmental Science and Technology*, 40(1):1–54, 2010

- Amend and Shock (2001): Jan P. Amend and Everett L. Shock. Energetics of overall metabolic reactions of thermophilic and hyperthermophilic archaea and bacteria. *FEMS Microbiology Reviews*, 25(2):175–243, 04 2001. ISSN 0168-6445. doi: 10.1111/j.1574-6976.2001.tb00576.x. URL `https://doi.org/10.1111/j.1574-6976.2001.tb00576.x`

- Stumm (1996): Werner Stumm, JJ Morgan, et al. Chemical equilibria and rates in natural waters. *Aquatic chemistry*, 1022, 1996

- eQuilibrator: Elad Noor, Hulda S. Haraldsdóttir, Ron Milo, and Ronan M. T. Fleming. Consistent estimation of gibbs energy using component contributions. *PLOS Computational Biology*, 9(7):1–11, 07 2013. doi: 10.1371/journal.pcbi.1003098

- NIST: Peter Linstrom and William Mallard. The nist chemistry webbook: A chemical data resource on the internet. (46), retrieved September 27, 2023

- Popovic (2019): Marko Popovic. Thermodynamic properties of microorganisms: determination and analysis of enthalpy, entropy, and gibbs free energy of biomass, cells and colonies of 32 microorganism species. *Heliyon*, 5 (6), 2019

- Naresh et al. (2011): Mohit Naresh, Sayoni Das, Prashant Mishra, and Aditya Mittal. The chemical formula of a magnetotactic bacterium. *Biotechnology and bioengineering*, 109(5):1205–1216, 2012

- Duboc et al. (1999) Philippe Duboc. Transient growth of saccharomyces cerevisiae. Technical report, EPFL, 1997

- Bauer and Ziv (1976): S. Bauer and E. Ziv. Dense growth of aerobic bacteria in a bench-scale fermentor. *Biotechnology and Bioengineering*, 18(1):81–94, 1976. doi: https://doi.org/10.1002/bit.260180107. URL `https://analyticalsciencejournals.onlinelibrary.wiley.com/doi/abs/10.1002/bit.260180107`

- Battley (1992): Edwin H Battley. Calculation of thermodynamic properties of protein in escherichia coli k-12 grown on succinic acid, energy changes accompanying protein anabolism, and energetic role of atp in protein synthesis. *Biotechnology and bioengineering*, 40(2):280–288, 1992

- van Dijken and Harder (1975): J. P. Van Dijken and W. Harder. Growth yields of microorganisms on methanol and methane. a theoretical study. *Biotechnology and Bioengineering*, 17(1):15–30, 1975. doi: https://doi.org/10.1002/bit.260170103. URL `https://analyticalsciencejournals.onlinelibrary.wiley.com/doi/abs/10.1002/bit.260170103`

- Shimizu et al. (1978): Tatsuo Shimizu, Tateo Furuki, Tetsuro Waki, and Kunisuke Ichikawa. Metabolic characteristics of denitrification by paracoccus denitrificans. *Miscellaneous*, 56(3):207, 1978

- Stouthamer (1977): AH Stouthamer and Corry W Bettenhaussen. A continuous culture study of an atpase-negative mutant of escherichia coli. *Archives of microbiology*, 113:185–189, 1977

- Mayberry et al. (1968): William Roy Mayberry, George John Prochazka, and William Jackson Payne. Growth yields of bacteria on selected organic compounds. *Applied Microbiology*, 15(6):1332–1338, 1967

- Battley, 1999a: Edwin H. Battley. An empirical method for estimating the entropy of formation and the absolute entropy of dried microbial biomass for use in studies on the thermodynamics of microbial growth. *Thermochimica Acta*, 326:7–15, 1999

- Harrison (1967): J.S. Harrison. Aspects of commercial yeast production. *Process Biochem.*, 2:41, 1967

- Kok and Roels (1980): Hermine E. de Kok and J. A. Roels. Method for the statistical treatment of elemental and energy balances with application to steady-state continuous-culture growth of saccharomyces cerevisiae cbs 426 in the respiratory region. *Biotechnology and Bioengineering*, 22(5):1097–1104, 1980. doi: https://doi.org/10.1002/bit.260220517. URL `https://analyticalsciencejournals.onlinelibrary.wiley.com/doi/abs/10.1002/bit.260220517`

- Wang et al. (2017): Henry Y. Wang, Duen-Gang Mou, and James R. Swartz. Thermodynamic evaluation of microbial growth. *Biotechnology and Bioengineering*, 18(12):1811–1814, 1976. doi: https://doi.org/10.1002/bit.260181214. URL `https://analyticalsciencejournals.onlinelibrary.wiley.com/doi/abs/10.1002/bit.260181214`

- Herbert (1976): D. Herbert. Stoichiometric aspects of microbial growth, 1976

- Wang et al. (2017): Li Wang, Xiaoyi Wang, Xuebo Jin, Jiping Xu, Huiyan Zhang, Jiabin Yu, Qian Sun, Chong Gao, and Lingbin Wang. Analysis of algae growth mechanism and water bloom prediction under the effect of multi-affecting factor. *Saudi Journal of Biological Sciences*, 24(3):556–562, 2017. ISSN 1319-562X. doi: https://doi.org/10.1016/j.sjbs.2017.01.026. URL `https://www.sciencedirect.com/science/article/pii/S1319562X17300359`. Computational Intelligence Research and Approaches in Bioinformatics and Biocomputing

- Manahan and Manahan (2009): S.E. Manahan S. Manahan. *Environmental Chemistry.* CRC Press, Boca Raton, 2009

- Phukan et al. (2011): Mayur M. Phukan, Rahul S. Chutia, B.K. Konwar, and R. Kataki. Microalgae chlorella as a potential bio-energy feedstock. *Applied Energy*, 88(10):3307–3312, 2011. ISSN 0306-2619. doi: https://doi.org/10.1016/j.apenergy.2010.11.026. URL `https://www.sciencedirect.com/science/article/pii/S0306261910004897`. Special Issue of Energy from algae: Current status and future trends

- Prajapati et al. (2014): Sanjeev Kumar Prajapati, Anushree Malik, and Virendra Kumar Vijay. Comparative evaluation of biomass production and bioenergy generation potential of chlorella spp. through anaerobic digestion. *Applied Energy*, 114:790–797, 2014. ISSN 0306-2619. doi: https://doi.org/10.1016/j.apenergy.2013.08.021. URL `https://www.sciencedirect.com/science/article/pii/S0306261913006569`

- Popovic et al. (2021): Marko Popovic, Gavin B.G. Stenning, Axel Göttlein, and Mirjana Minceva. Elemental composition, heat capacity from 2 to 300 k and derived thermodynamic functions of 5 microorganism species. *Journal of Biotechnology*, 331:99–107, 2021. ISSN 0168-1656

- Yamagata (1934): Syunzi Yamagata. *Über die elementare Zusammensetzung des Schimmelpilzkörpers.* 1934

- Battley (1987): Edwin H Battley. *Energetics of microbial growth.* Wiley, 1987

- Whelton and Doudoroff (1945): Rita Whelton and Michael Doudoroff. Assimilation of glucose and related compounds by growing cultures of pseudomonas saccharophila. *Journal of Bacteriology*, 49(2):177–186, 1945

- Samejima and Meyers (1958): H Samejima and J Myers. On the heterotrophic growth of chlorella pyrenoidosa. *Microbiology*, 18(1):107–117, 1958

- Hoover and Allison (1940): Sam R Hoover and Franklin E Allison. The growth metabolism of rhizobium, with evidence on the interrelations between respiration and synthesis. *Journal of Biological Chemistry*, 134(1):181–192, 1940

- Tamiya and Usami (1939): from Edwin H Battley. *Energetics of microbial growth.* Wiley, 1987

- Traore et al. (1981): from Edwin H Battley. *Energetics of microbial growth.* Wiley, 1987

- Liu at al. (2001): J-S Liu, IW Marison, and U Von Stockar. Microbial growth by a net heat up-take: a calorimetric and thermodynamic study on acetotrophic methanogenesis by methanosarcina barkeri. *Biotechnology and bioengineering*, 75(2):170–180, 2001

- Schill et al. (1999): Natascha A Schill, Jing-Song Liu, and Urs von Stockar. Thermodynamic analysis of growth of methanobacterium thermoautotrophicum. *Biotechnology and bioengineering*, 64(1):74–81, 1999

- Cordier et al. (1987): Jean-Louis Cordier, Bertram M Butsch, Bernard Birou, and Uros von Stockar. The relationship between elemental composition and heat of combustion of microbial biomass. *Applied Microbiology and Biotechnology*, 25:305–312, 1987

- Andersen (1980): Klaus B Andersen and Kaspar von Meyenburg. Are growth rates of escherichia coli in batch cultures limited by respiration? *Journal of bacteriology*, 144(1):114–123, 1980

- Badziong (1978): Werner Badziong and Rudolf K Thauer. Growth yields and growth rates of desulfovibrio vulgaris (marburg) growing on hydrogen plus sulfate and hydrogen plus thiosulfate as the sole energy sources. *Archives of microbiology*, 117:209–214, 1978

- Badziong (1978)a: Werner Badziong, Rudolf K Thauer, and J Gregory Zeikus. Isolation and characterization of desulfovibrio growing on hydrogen plus sulfate as the sole energy source. *Archives of Microbiology*, 116:41–49, 1978
- Brandis (1981): Astrid Brandis and Rudolf K Thauer. Growth of desulfovibrio species on hydrogen and sulphate as sole energy source. *Microbiology*, 126(1):249–252, 1981
- Chua (1983): HB Chua and JP Robinson. Formate-limited growth of methanobacterium formicium in steady-state cultures. *Archives of microbiology*, 135:158–160, 1983
- Clarens (1990): M Clarens and R Molleta. Kinetic studies of acetate fermentation by methanosarcina sp. msta-1. *Applied microbiology and biotechnology*, 33:239–244, 1990
- Crabbendam (1985): Pia M Crabbendam, OM Neijssel, and DW Tempest. Metabolic and energetic aspects of the growth of clostridium butyricum on glucose in chemostat culture. *Archives of microbiology*, 142:375–382, 1985
- Dijkhuizen (1977): L Dijkhuizen, M Wiersma, and W Harder. Energy production and growth of pseudomonas oxalaticus ox1 on oxalate and formate. *Archives of Microbiology*, 115:229–236, 1977
- Esteve-Nunez (2005): Abraham Esteve-Núñez, Mary Rothermich, Manju Sharma, and Derek Lovley. Growth of geobacter sulfurreducens under nutrient-limiting conditions in continuous culture. *Environmental microbiology*, 7(5):641–648, 2005
- Goldberg (1976): I Goldberg, JS Rock, A Ben-Bassat, and RI Mateles. Bacterial yields on methanol, methylamine, formaldehyde, and formate. *Biotechnology and bioengineering*, 18(12):1657–1668, 1976
- Hernandez (1967): Eovaldo Hernandez and Marvin J Johnson. Energy supply and cell yield in aerobically grown microorganisms. *Journal of Bacteriology*, 94(4):996–1001, 1967
- Heyndrickx (1991): M Heyndrickx, P De Vos, and J De Ley. Fermentation characteristics of clostridium pasteurianum lmg 3285 grown on glucose and mannitol. *Journal of Applied Microbiology*, 70(1):52–58, 1991
- Huser (1982): Beat A Huser, Karl Wuhrmann, and Alexander JB Zehnder. Methanothrix soehngenii gen. nov. sp. nov., a new acetotrophic non-hydrogen-oxidizing methane bacterium. *Archives of Microbiology*, 132:1–9, 1982
- Ingvorsen (1984) 2: Kjeld Ingvorsen, Alexander JB Zehnder, and Bo B Jørgensen. Kinetics of sulfate and acetate uptake by desulfobacter postgatei. *Applied and environmental microbiology*, 47(2):403–408, 1984
- Laanbroek (1994): Hendrikus J Laanbroek, Harm J Geerligs, Lolke Sijtsma, and Hans Veldkamp. Competition for sulfate and ethanol among desulfobacter, desulfobulbus, and desulfovibrio species isolated from intertidal sediments. *Applied and Environmental Microbiology*, 47(2):329–334, 1984
- Lin (2007): Pei-Ying Lin, Liang-Ming Whang, Yi-Ru Wu, Wei-Jie Ren, Chia-Jung Hsiao, Shiue-Lin Li, and Jo-Shu Chang. Biological hydrogen production of the genus clostridium: metabolic study and mathematical model simulation. *International Journal of Hydrogen Energy*, 32(12):1728–1735, 2007
- Lovitt (1987): RW Lovitt, DB Kell, and JG Morris. The physiology of clostridium sporogenes ncib 8053 growing in defined media. *Journal of applied bacteriology*, 62(1):81–92, 1987
- Mayberry (1967): William Roy Mayberry, George John Prochazka, and William Jackson Payne. Growth yields of bacteria on selected organic compounds. *Applied Microbiology*, 15(6):1332–1338, 1967
- Myers (1988): Charles R Myers and Kenneth H Nealson. Bacterial manganese reduction and growth with manganese oxide as the sole electron acceptor. *Science*, 240(4857):1319–1321, 1988
- Patel (1984): GB Patel. Characterization and nutritional properties of methanothrix concilii sp. nov., a mesophilic, aceticlastic methanogen. *Canadian Journal of Microbiology*, 30(11):1383–1396, 1984
- Peters (1998): V Peters, PH Janssen, and R Conrad. Efficiency of hydrogen utilization during unitrophic and mixotrophic growth of acetobacterium woodii on hydrogen and lactate in the chemostat. *FEMS Microbiology Ecology*, 26(4):317–324, 1998

- Pfennig (1976): Norbert Pfennig and Hanno Biebl. Desulfuromonas acetoxidans gen. nov. and sp. nov., a new anaerobic, sulfur-reducing, acetate-oxidizing bacterium. *Archives of Microbiology*, 110:3–12, 1976
- Robinson (1984): Joseph A Robinson and James M Tiedje. Competition between sulfate-reducing and methanogenic bacteria for h 2 under resting and growing conditions. *Archives of Microbiology*, 137:26–32, 1984
- Roden (1993): Eric E Roden and Derek R Lovley. Dissimilatory fe (iii) reduction by the marine microorganism desulfuromonas acetoxidans. *Applied and Environmental Microbiology*, 59(3):734–742, 1993
- Rutgers (1989): Michiel Rutgers, Hanneke ML van der Gulden, and Karelvan Dam. Thermodynamic efficiency of bacterial growth calculated from growth yield of pseudomonas oxalaticus ox1 in the chemostat. *Biochimica et Biophysica Acta (BBA)-Bioenergetics*, 973(2):302–307, 1989
- Sanford (2007): Robert A Sanford, Qingzhong Wu, Youlboong Sung, Sara H Thomas, Benjamin K Amos, Emily K Prince, and Frank E Löffler. Hexavalent uranium supports growth of anaeromyxobacter dehalogenans and geobacter spp. with lower than predicted biomass yields. *Environmental microbiology*, 9(11):2885–2893, 2007
- Sass (2004): Henrik Sass, Jörg Overmann, Heike Rütters, Hans-Dietrich Babenzien, and Heribert Cypionka. Desulfosporomusa polytropa gen. nov., sp. nov., a novel sulfate-reducing bacterium from sediments of an oligotrophic lake. *Archives of microbiology*, 182:204–211, 2004
- Schauer (1980): Neil L Schauer and James G Ferry. Metabolism of formate in methanobacterium formicicum. *Journal of Bacteriology*, 142(3):800–807, 1980
- Smith (1978): Michael R Smith and Robert A Mah. Growth and methanogenesis by methanosarcina strain 227 on acetate and methanol. *Applied and Environmental Microbiology*, 36(6):870–879, 1978
- Zehnder (1977): AJB Zehnder and K Wuhrmann. Physiology of a methanobacterium strain az. *Archives of Microbiology*, 111:199–205, 1977
- Weimer (1978): PJ Weimer and JG Zeikus. One carbon metabolism in methanogenic bacteria: cellular characterization and growth of methanosarcina barkeri. *Archives of Microbiology*, 119:49–57, 1978
- Stieb (1989): Marion Stieb and Bernhard Schink. Anaerobic degradation of isobutyrate by methanogenic enrichment cultures and by a desulfococcus multivorans strain. *Archives of microbiology*, 151:126–132, 1989
- Strohm (2007): Tobin O Strohm, Ben Griffin, Walter G Zumft, and Bernhard Schink. Growth yields in bacterial denitrification and nitrate ammonification. *Applied and environmental microbiology*, 73(5):1420–1424, 2007
- Szewzyk (1990): Regine Szewzyk and Norbert Pfennig. Competition for ethanol between sulfate-reducing and fermenting bacteria. *Archives of microbiology*, 153:470–477, 1990
- Tang (2007): Yinjie J Tang, Adam L Meadows, and Jay D Keasling. A kinetic model describing shewanella oneidensis mr-1 growth, substrate consumption, and product secretion. *Biotechnology and bioengineering*, 96 (1):125–133, 2007
- Vasiliadou (2006): IA Vasiliadou, S Pavlou, and DV Vayenas. A kinetic study of hydrogenotrophic denitrification. *Process biochemistry*, 41(6):1401–1408, 2006
- Wallrabenstein (1995): Christina Wallrabenstein, Elisabeth Hauschild, and Bernhard Schink. Syntrophobacter pfennigii sp. nov., new syntrophically propionate-oxidizing anaerobe growing in pure culture with propionate and sulfate. *Archives of Microbiology*, 164:346–352, 1995
- Widdel (1977): Friedrich Widdel and Norbert Pfennig. A new anaerobic, sporing, acetate-oxidizing, sulfate-reducing bacterium, desulfotomaculum (emend.) acetoxidans. *Archives of Microbiology*, 112:119–122, 1977
- Widdel (1981): Friedrich Widdel and Norbert Pfennig. Studies on dissimilatory sulfate-reducing bacteria that decompose fatty acids: I. isolation of new sulfate-reducing bacteria enriched with acetate from saline environments. description of desulfobacter postgatei gen. nov., sp. nov. *Archives of microbiology*, 129:395–400, 1981
- Widdel (1982): Friedrich Widdel and Norbert Pfennig. Studies on dissimilatory sulfate-reducing bacteria that decompose fatty acids ii. incomplete oxidation of propionate by desulfobulbus propionicus gen. nov., sp. nov. *Archives of Microbiology*, 131:360–365, 1982

- Yang (1987): Shang-Tian Yang and MR Okos. Kinetic study and mathematical modeling of methanogenesis of acetate using pure cultures of methanogens. *Biotechnology and bioengineering*, 30(5):661–667, 1987
- Yoon (2013): Sukhwan Yoon, Robert A Sanford, and Frank E Löffler. Shewanella spp. use acetate as an electron donor for denitrification but not ferric iron or fumarate reduction. *Applied and environmental microbiology*, 79 (8):2818–2822, 2013
- de Poorter (2007): Linda MI De Poorter, Wim J Geerts, and Jan T Keltjens. Coupling of methanothermobacter thermautotrophicus methane formation and growth in fed-batch and continuous cultures under different h2 gassing regimens. *Applied and environmental microbiology*, 73(3):740–749, 2007
- de Vries (1980): Wytske de Vries, HGD Niekus, Marian Boellaard, and AH Stouthamer. Growth yields and energy generation by campylobacter sputorum subspecies bubulus during growth in continuous culture with different hydrogen acceptors. *Archives of Microbiology*, 124:221–227, 1980
- Birou (1987): Bernard Birou, Ian W Marison, and Urs Von Stockar. Calorimetric investigation of aerobic fermentations. *Biotechnology and bioengineering*, 30(5):650–660, 1987
- Stockar (1999): U Von Stockar and J-S Liu. Does microbial life always feed on negative entropy? thermodynamic analysis of microbial growth. *Biochimica et Biophysica Acta (BBA)-Bioenergetics*, 1412(3):191–211, 1999
- Brettel (1980): R Brettel, I Lamprecht, and B Schaarschmidt. Microcalorimetric investigations of the metabolism of yeasts vii. flow-calorimetry of aerobic batch cultures. *Radiation and Environmental Biophysics*, 18:301–309, 1980
- Dejean (2000): Laurent Dejean, Bertrand Beauvoit, Bernard Guérin, and Michel Rigoulet. Growth of the yeast saccharomyces cerevisiae on a non-fermentable substrate: control of energetic yield by the amount of mitochondria. *Biochimica et Biophysica Acta (BBA)-Bioenergetics*, 1457(1-2):45–56, 2000
- Battley (1960): Edwin H Battley. Enthalpy changes accompanying the growth of saccharomyces cerevisiae (hansen). *Physiologia Plantarum*, 13(4):628–640, 1960
- Battley (1998): Edwin H Battley. The development of direct and indirect methods for the study of the thermodynamics of microbial growth. *Thermochimica Acta*, 309(1-2):17–37, 1998
- Dermoun (1979): Z Dermoun and JP Belaich. Microcalorimetric study of escherichia coli aerobic growth: kinetics and experimental enthalpy associated with growth on succinic acid. *Journal of Bacteriology*, 140(2): 377–380, 1979
- Dermoun (1985): Z Dermoun and JP Belaich. Microcalorimetric study of cellulose degradation by cellulomonas uda atcc 21399. *Biotechnology and bioengineering*, 27(7):1005–1011, 1985
- Belaich (1976): A Belaich and JP Belaich. Microcalorimetric study of the anaerobic growth of escherichia coli: growth thermograms in a synthetic medium. *Journal of bacteriology*, 125(1):14–18, 1976
- Ishikawa (1981): Yasufumi Ishikawa, Yukio Nonoyama, and Makoto Shoda. Microcalorimetric study of aerobic growth of escherichia coli in batch culture. *Biotechnology and Bioengineering*, 23(12):2825–2836, 1981
- Ishikawa (1983): Yasufumi Ishikawa and Makoto Shoda. Calorimetric analysis of escherichia coli in continuous culture. *Biotechnology and Bioengineering*, 25(7):1817–1827, 1983
- Tamiya (1935): H Tamiya. Material and energy balances of biological synthesis. *Actualities Scientifiques et Industrielles*, 214, 1935
- Whelton (1945): Rita Whelton and Michael Doudoroff. Assimilation of glucose and related compounds by growing cultures of pseudomonas saccharophila. *Journal of Bacteriology*, 49(2):177–186, 1945
- Samejima (1958): H Samejima and J Myers. On the heterotrophic growth of chlorella pyrenoidosa. *Microbiology*, 18(1):107–117, 1958
- Hoover (1940): Sam R Hoover and Franklin E Allison. The growth metabolism of rhizobium, with evidence on the interrelations between respiration and synthesis. *Journal of Biological Chemistry*, 134(1):181–192, 1940

- Liu (2001): J-S Liu, IW Marison, and U Von Stockar. Microbial growth by a net heat up-take: a calorimetric and thermodynamic study on acetotrophic methanogenesis by methanosarcina barkeri. *Biotechnology and bioengineering*, 75(2):170–180, 2001
- Birou (1989): Bernard Birou and Urs Von Stockar. Application of bench-scale calorimetry to chemostat cultures. *Enzyme and microbial technology*, 11(1):12–16, 1989
- Marison (1987): Ian Marison and Urs Von Stockar. A calorimetric investigation of the aerobic cultivation of kluyveromyces fragilis on various substrates. *Enzyme and microbial technology*, 9(1):33–43, 1987
- Patino (2007): Rodrigo Patino, Marcel Janssen, and Urs von Stockar. A study of the growth for the microalga chlorella vulgaris by photo-bio-calorimetry and other on-line and off-line techniques. *Biotechnology and bioengineering*, 96(4):757–767, 2007
- Zhao (2004): J Zhao, T Baba, H Mori, and K Shimizu. Global metabolic response of escherichia coli to gnd or zwf gene-knockout, based on 13 c-labeling experiments and the measurement of enzyme activities. *Applied microbiology and biotechnology*, 64:91–98, 2004
- Hempfling (1975): Walter P Hempfling and Stanley E Mainzer. Effects of varying the carbon source limiting growth on yield and maintenance characteristics of escherichia coli in continuous culture. *Journal of bacteriology*, 123(3):1076–1087, 1975
- Mainzer (1976): Stanley E Mainzer and Walter P Hempfling. Effects of growth temperature on yield and maintenance during glucose-limited continuous culture of escherichia coli. *Journal of bacteriology*, 126(1):251–256, 1976
- Verduyn thesis, in Heijnen (1992): JJ Heijnen and JP Van Dijken. In search of a thermodynamic description of biomass yields for the chemotrophic growth of microorganisms. *Biotechnology and Bioengineering*, 39(8):833–858, 1992
- Verduyn thesis: Cornelis Verduyn. Energetic aspects of metabolic fluxes in yeasts. 1992
- Bernacchi (2014): Sébastien Bernacchi, Simon Rittmann, Arne H Seifert, Alexander Krajete, and Christoph Herwig. Experimental methods for screening parameters influencing the growth to product yield (y (x/ch4)) of a biological methane production (bmp) process performed with methanothermobacter marburgensis. *AIMS Bioengineering*, 1(2):72–87, 2014
- Liu (1999): Jing-Song Liu, Natascha Schill, Walter M van Gulik, Damien Voisard, Ian W Marison, and Urs von Stockar. The coupling between catabolism and anabolism of methanobacterium thermoautotrophicum in h2-and iron-limited continuous cultures. *Enzyme and microbial technology*, 25(10):784–794, 1999
- Schill (1999): Natascha A Schill, Jing-Song Liu, and Urs von Stockar. Thermodynamic analysis of growth of methanobacterium thermoautotrophicum. *Biotechnology and bioengineering*, 64(1):74–81, 1999
